# Energy landscape of conformational changes for a single unmodified protein

**DOI:** 10.1038/s44328-024-00014-x

**Published:** 2024-11-06

**Authors:** Matthew Peters, Tianyu Zhao, Sherin George, Viet Giang Truong, Síle Nic Chormaic, Cuifeng Ying, René A. Nome, Reuven Gordon

**Affiliations:** 1 Department of Electrical Engineering, University of Victoria, Victoria, V8W 2Y2 BC Canada; 2 Center for Advanced Material & Related Technologies, University of Victoria, Victoria, V8W 2Y2 BC Canada; 3 Okinawa Institute of Science and Technology Graduate University, Onna, Okinawa 904-0495 Japan; 4 Advanced Optics and Photonics Laboratory, Department of Engineering, School of Science & Technology, Nottingham Trent University, Nottingham, NG11 8NS England; 5 Institute of Chemistry, State University of Campinas, Campinas, Brazil

**Keywords:** Optical techniques, Proteins, Biophysics, Molecular biophysics

## Abstract

Resolving the free energy landscapes that govern protein biophysics has been obscured by ensemble averaging. While the folding dynamics of single proteins have been observed using fluorescent labels and/or tethers, a simpler and more direct measurement of the conformational changes would not require modifications to the protein. We use nanoaperture optical tweezers to resolve the energy landscape of a single unmodified protein, Bovine Serum Albumin (BSA), and quantify changes in the three-state conformation dynamics with temperature. A Markov model with Kramers’ theory transition rates is used to model the dynamics, showing good agreement with the observed state transitions. This first look at the intrinsic energy landscape of proteins provides a transformative tool for protein biophysics and may be applied broadly, including mapping out the energy landscape of particularly challenging intrinsically disordered proteins.

## Introduction

In the physiological environment, proteins exist not as single rigid structures, but as dynamic entities sampling many conformations across a free-energy landscape. At the most basic level, the free-energy landscape is a graph of energy across a reaction coordinate that underlies the thermodynamics, and at the single-molecule level, the molecular biophysics. Conformational dynamics are a common example of trajectories on the energy landscape, largely responsible for protein function. To observe these dynamics, single-molecule approaches are favoured because they do not require synchronisation and naturally allow for observing heterogeneity^1^.

Common approaches to single molecule investigations of proteins employ labels (e.g., in Förster resonance energy transfer)^2–4^ or tethers (e.g., in force-measurement techniques)^5–11^. While these have been highly successful, they are not without challenges in terms of observation time and energy scale (e.g., pulling measurements apply forces that completely unravel the protein). These techniques also require the introduction of a label or tether (with additional steps, more hardware, and perhaps modifying the intrinsic function of the protein^12–19^). Therefore, approaches that can directly observe unmodified protein conformation dynamics at the single molecule level are desired. While many approaches are emerging to observe unmodified proteins (label-free, tether-free)^20–31^, so far the conformational dynamics of a single unmodified protein remain largely unexplored.

Nanoaperture optical tweezers (NOTs) have been employed by many groups to observe biomolecules and nanoparticles at the single molecule level without the need for any modification^21,24,32–47^. The first work to demonstrate the trapping of a single protein with NOTs used Bovine Serum Albumin (BSA) as a model system^34^. In that work, the conformational changes of BSA were seen by changes in the optical transmission of the trapping laser beam through the nanoaperture, where transitions from the normal to fast form of the protein resulted in an increase in transmission. This was confirmed by lowering the pH and forcing the BSA into the fast form so that only the larger transmission state was observed. However, quantifying the observed dynamics and using them to determine the energy landscape was not shown in that work. A direct measurement of a single protein’s energy landscape can lead to a better understanding of thermodynamic properties, allostery, intrinsically disordered proteins and prion diseases, to name a few examples.

Here we show the connection between the probability density function (PDF) of the trapping laser’s scattering and the energy landscape along the protein conformation reaction coordinate. We produce the free-energy landscape governing normal (N), fast (F), and expanded (E) conformations of BSA, mapping out changes in the landscape with temperature. We quantify the thermodynamics of these transitions, finding the entropy change associated with a transition and using a Markov model with Kramers’ transition rate probabilities to capture the observed dynamics. We also observe dimer formation in the trap (directly seen when a second BSA enters the trap). We show that the dimer formation has a stabilising effect, suppressing F and E forms, while we quantify the open (O) and closed (C) hinge transitions of the dimer.

Our work follows a large body of literature on BSA. BSA is a 66 kDa water-soluble monomeric protein that is the most abundant protein in blood (40-50 mg/mL). Its structure is well studied and the conformational changes are largely explained by changes in the disulphide bridges of the Cys residues^48–52^. Adjacent Cys residues are unable to form disulphide bridges because the highly favoured trans-peptide bond during folding prevents the sulfhydryl groups from being close enough to bond. Thus, the adjacent Cys residues must bridge to Cys residues further away. Three domains emerge where the polypeptide backbone is joined by the double-Cys bridge (domains I, II, II). These three domains are similar, formed by six *α*-helices arranged in a heart shape with 17 disulphide bond linkages stabilising the domains and one sulfhydryl group (Cys-34, domain I, hydrophobic pocket). Each domain is composed of two subdomains (A and B). BSA has dimensions 80 × 80 × 40 Å^3^ and is composed of 583 amino acid residues. Key to the function of BSA are the hydrophobic pockets that are not exposed to water in the N form, but are exposed in the F and E forms. At neutral pH, the disulphide bridges are buried in the protein and not exposed to solvent.

BSA conformations have typically been probed using pH changes^53–57^ and temperature changes^58–61^. These conformational changes are reversible (provided the temperature is below 55 °C^62,63^). At room temperature and neutral pH, BSA exists in the N-state (heart shape), and at pH 3.5, it undergoes a conformational transformation into a more linear shape F-state. Three independent folded conformations have been observed by smFRET and differential scanning calorimetry^64,65^, with the E state being the most expanded. To remain consistent in the terminology used with pH experiments, we define the three states observed in our experiments as N, F, and E, indicating native, expanded due to domain III opening, and maximally expanded due to domain I and III opening.

## Results

### Experimental configuration

**Fig. 1:**
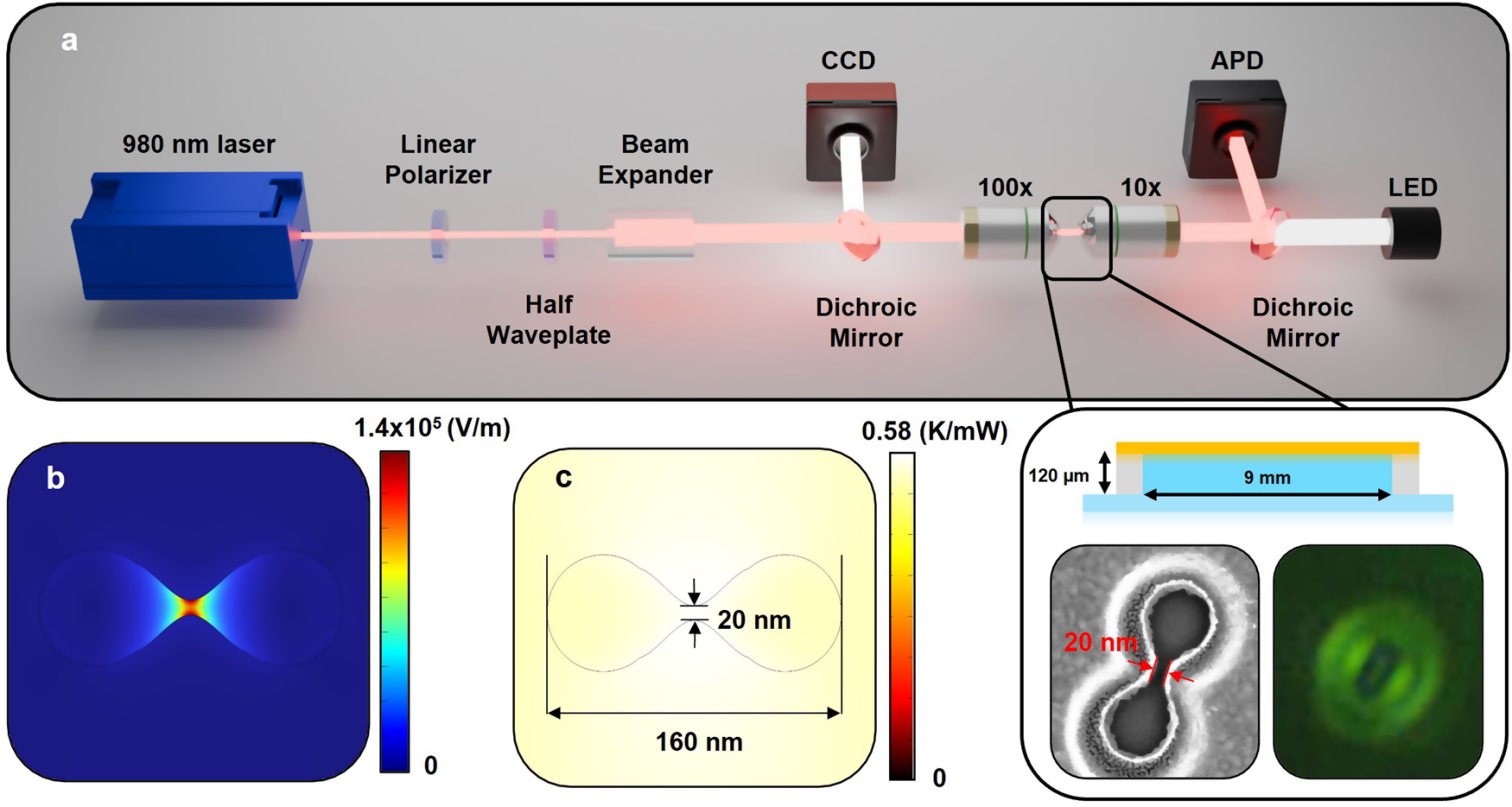
Experimental configuration and COMSOL multiphysics simulations for nanoaperture optical trapping. **a** Simplified microscope diagram. Laser passes through a linear polarizer and half-waveplate to control polarisation orientation and a beam expander to fill the objective. A 100 × 1.25 NA oil immersion objective is incident on the sample and transmitted light is collected by a 10 × 0.25 NA objective and recorded on an avalanche photodiode (APD). Inset: Schematic of microwell used for trapping solution and SEM and camera image of a DNH used in experiments. Camera image is under focused to highlight the oval shape of the DNH and represent the focus state during trapping. **b** Electric field and (**c**) thermal distribution simulated by COMSOL Multiphysics for a DNH in aqueous using incident 980 nm laser with 1 mW power and beam diameter of 1 μm.

Details of the experiment are presented in the Methods section. Briefly, a simplified schematic of the optical tweezers is shown in Fig. 1a. A schematic of the microwell, an electron microscope image of a double nanohole (DNH), and an image of a DNH on the camera is seen in the expanded view. Simulations of the electric field enhancement and thermal distribution with a 980 nm incident laser are shown in Fig. 1b, c. The local temperature increase due to heating from an incident laser on a nanoaperture has been studied in multiple works^42,66–68^, with values reported from 0.6 K/mW to 3.6 K/mW. We performed COMSOL simulations of the local heating and estimated 0.58 K/mW of laser power as defined after the microscope objective (see Fig. SI-1a, b) In past experiments on a nominally identical setup, we used ratiometric analysis of upconverter emission to determine the local temperature changes with laser power, and found 0.64 K/mW was the heating^68^. The local temperature at the protein was varied by changing the trapping laser power.

### Protein dynamics

**Fig. 2:**
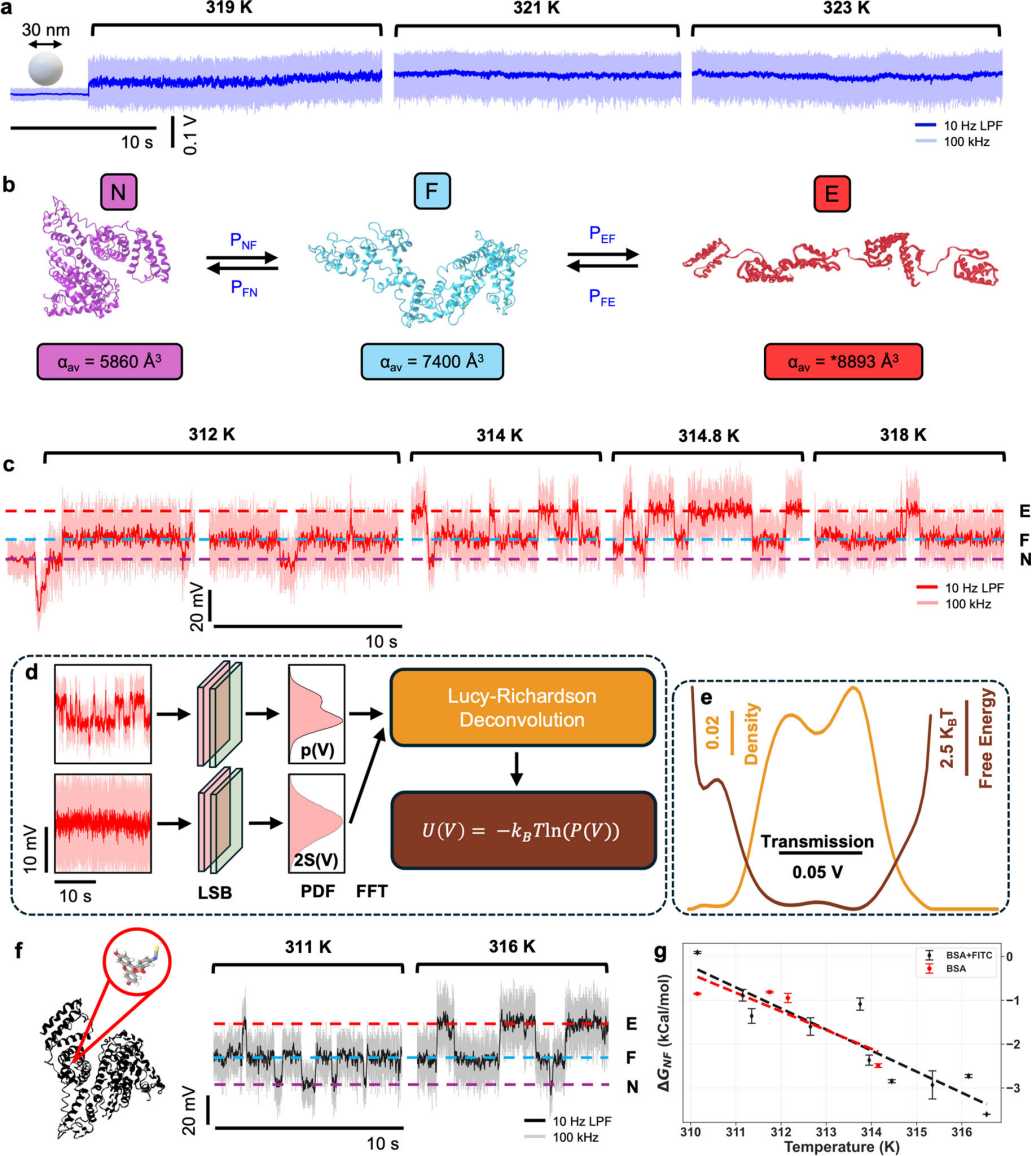
Nanoaperture optical trapping transmission traces of 30 nm polystyrene and BSA for increasing temperatures and corresponding energy landscapes. **a** Transmission through the DNH at different temperatures for a 30 nm polystyrene nanoparticle trap. Dark blue indicates data filtered with a 10 Hz low-pass filter, light blue indicates data sampled at 100 kHz. **b** Reaction mechanism diagram for the forward and reverse conformational changes of N ↔ F and F ↔ E and the corresponding average molecular polarizabilities. Proteins visualised using PDB: 3V03 for the N-state and PDB: “F isoform of BSA at pH 3.5^49^” for the F-state. No 3D structure is available for the E-state, illustration is traced past work^77^. Polarizability estimated from experimental data. **c** Transmission through the DNH measured at different temperatures for a single unlabelled BSA trap. Dark red indicates data filtered with a 10 Hz low-pass filter, light red is raw data at 100 kHz. **d** Deconvolution of transmission probability density function (100 bins, 100 kHz sampling rate) and PSF to obtain the energy landscape. LSB least significant bit dithering, PDF probability density function. **e** Energy landscape and probability density function after deconvolution for unlabelled BSA at 314 K. **f** Transmission through the DNH measured at different temperatures for a single FITC labelled BSA trap. Black indicates data filtered with 10 Hz low-pass filter, grey is raw data at 100 kHz. **g** Change in Gibbs free energy for unlabelled and labelled BSA, obtained from the energy landscape at different temperatures.

Conformational changes of a protein alter the structure, and thus the polarizability^69^. This changes the transmission through the DNH. As a control experiment, Fig. 2a shows a trapping event of a 30 nm diameter polystyrene nanoparticle that does not change conformation upon trapping at different temperatures. We expect that the trapping position for PS nanoparticles is the same as BSA because of the larger sized gap used. The transmission is stable for all temperatures as there is no structural rearrangement, nor is there evidence that the translational motion in the trap would result in discrete levels of the signal that we present next for conformational changes. Since the temperature increase is attained by increasing the laser power, the signal to noise ratio also increases (signal increases linearly with laser power and noise as the square root, since we are in the shot-noise limited regime).

Figure 2b shows the N- and F-state of monomeric BSA with average molecular polarizabilities calculated to be 5860 Å^3^ (PDB: 3V03) and 7400 Å^3^ (PDB: F isoform of BSA at pH 3.5^49^) using an atomistic model of the protein^69^ and visualised using iCn3D^70^. There is no PDB molecular structure available for the E-state; however, the polarizability can be estimated at 8893 Å^3^, as extrapolated from the root mean square deviation of the signal for the laser, N, and F states (see Supplementary Information Fig SI-3 and Table SI-1).

Conformational changes of a single BSA protein are easily identified as changes in transmission through the DNH. Figure 2c shows the effect of temperature increase on the dynamics of a single BSA. The dip in transmission signal is seen in all trapping events for both proteins and polystyrene nanoparticles, this is due to the scattering phase change around the aperture leading to destructive interference during the initial stage of trapping. The protein is initially trapped in the N-state, as expected, and quickly transitions to the F-state. The transitions at 312 K mostly fluctuate between F- and N-, with a rare E- occurrence. As the temperature is increased, the rate of N ↔ F and F ↔ E transitions shifts to favour the more expanded form.

The energy landscape of these conformational changes can be obtained from the PDF, a binned histogram of the APD voltage recorded for an extended time-window. For force-measurement approaches, the deconvolution of a point spread function (PSF) has been widely investigated as the tethers are fluctuating separately from the conformational dynamics of the protein^9^. Here, the translational or rotational motion of the protein will give fluctuations to the signal that are distinct from conformational changes. These fluctuations (and other factors such as noise) give a PSF to the signal that should be deconvolved to extract the true PDF associated with the conformational changes and from this, the energy landscape. The energy landscape and PSF are determined from the raw data sampled at 100 kHz. Filtering of the data necessarily constrains the trapping signal into the region of the energy landscape minima, artificially changing the extracted biophysical parameters, and so this is not done when producing the energy landscape.

For rigid structures, the distribution from translational motion is well-fit by a Gaussian, with FWHM linearly proportional to the protein molecular weight, as found previously for multiple proteins^71^. Using this information, we can estimate a PSF, *C*(*V*), for our BSA measurements to have a standard deviation of 13.4 mV, which we can determine from calibration curves like in past works^71^, but here we get it from the dimer due to the enhanced conformational stability allowing for longer observation without a transition. We investigated the impact of small variations on the robustness of this PSF and found little variation within 1.2 mV deviations.

The PDF of the raw transmission data, *p*(*V*), was deconvolved with the Gaussian PSF using the iterative Lucy-Richardson algorithm to uncover the energy landscape *P*(*V*):

1 $$P{(V)}^{(k+1)}=P{(V)}^{(k)}\left[\frac{p(V)}{P{(V)}^{(k)}\otimes C(V)}\otimes C{(V)}^{* }\right]$$

where *k* is the iteration number, ⊗ denotes convolution, and ${*}$ denotes complex conjugate, with *P*(*V*)^(1)^ = *p*(*V*). *P*(*V*) = *P*(*V*)^*M*^, with *M* = 51 being the total number of iterations to achieve convergence.

The energy landscape was thus obtained by the following equation:

2 $$U(V)=-{k}_{B}T\ln (P(V)),$$

where *k*_*B*_ is the Boltzmann constant and *T* is the temperature. A schematic overview of this process is shown in Fig. 2d and the resulting PDF and energy landscape of BSA at 314 K is shown in Fig. 2e.

We repeated the measurement with fluorescein isothiocyanate (FITC) labelled BSA to see if different results would be obtained for the labelled form. FITC binds to site II of BSA, filling an empty hydrophobic site of domain III, noting the FITC itself is also hydrophobic. A schematic of FITC-BSA and a transmission time trace at different temperatures is shown in Fig. 2f. The change in Gibbs free energy for both labelled and unlabelled data is shown in Fig. 2g, demonstrating a temperature dependence on the spontaneity of the conformational changes and equilibria of states.

**Fig. 3:**
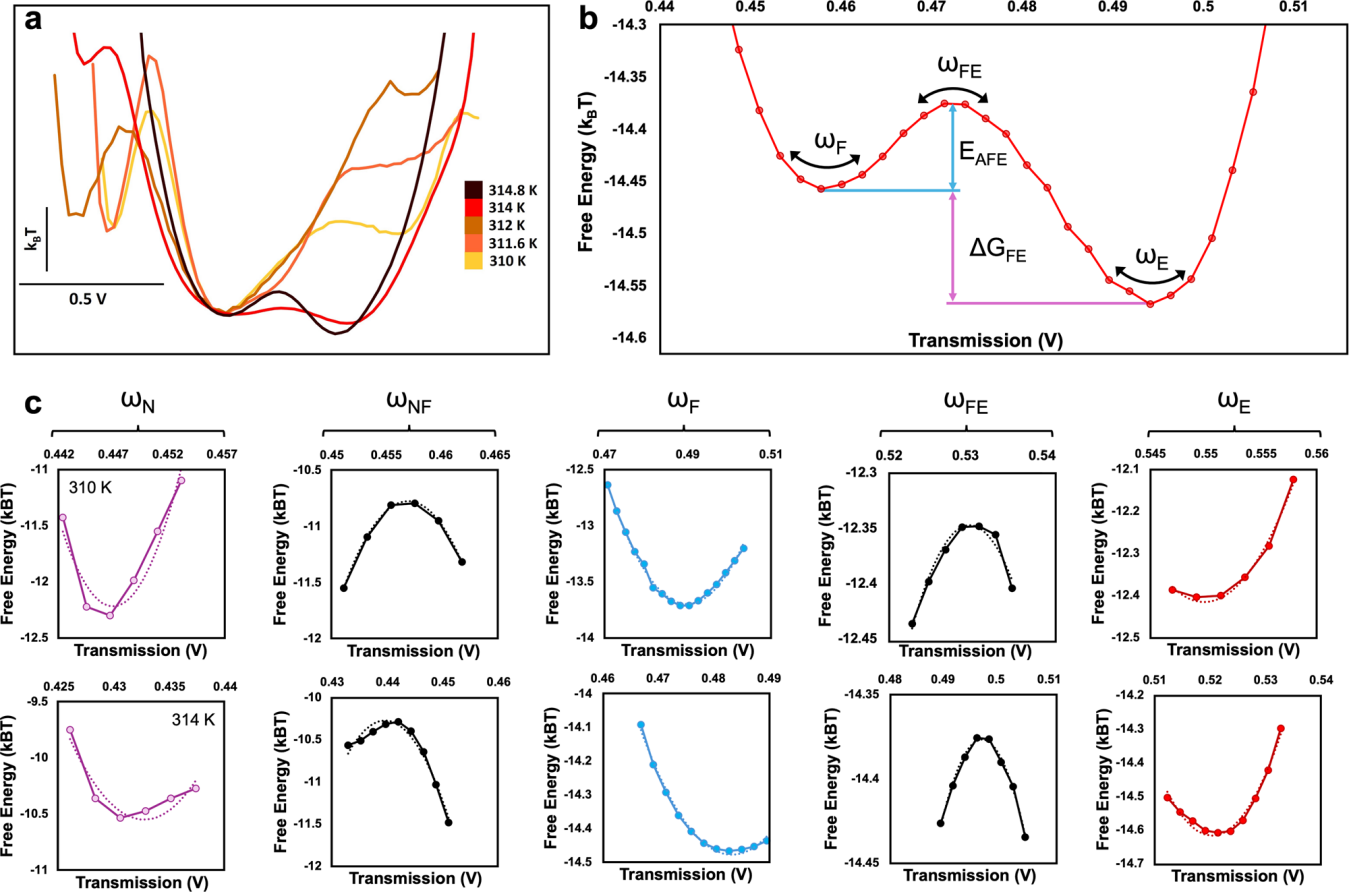
Quantitative assessment of energy landscapes obtained from the trapping transmission traces. **a** Energy landscapes for trapping events at increasing temperatures. **b** Example of quantitative values obtained from the energy landscape, where *ω* is the curvature, *E*_*AFE*_ is the activation energy for the FE transition, and Δ*G*_*FE*_ is the change in Gibb’s free energy of the FE transition. **c** Curvatures from the energy landscape fit to a second order polynomial at 310 K and 314 K.

The energy landscape evolution with temperature is shown in Fig. 3a. The energy minima shows an evolution towards E and F states, and away from N state as the temperature increases, as noted by changes in the relative Gibbs free energies of these minima. Quantitative values are obtained from the landscape by measuring free energy differences between states and the curvatures of the wells and barriers, as shown in Fig. 3b. Examples of well and barrier curvatures for two temperatures, 310 K and 314 K are shown in Fig. 3c.

The thermodynamic equilibrium constant, *K*, is the quotient of the activity of the reaction and is dependent only on temperature, *T*. A large *K* value indicates a highly favoured product, or in this case, a favoured conformation. This follows the equation:

3 $${K}_{eq}={e}^{-\frac{\Delta G}{RT}}.$$

where Δ*G* is the change in Gibbs free energy and *R* is the gas constant. We calculate *K*_*eq*_ using the minima in the energy landscape, with more detail found in the Methods section.

**Fig. 4:**
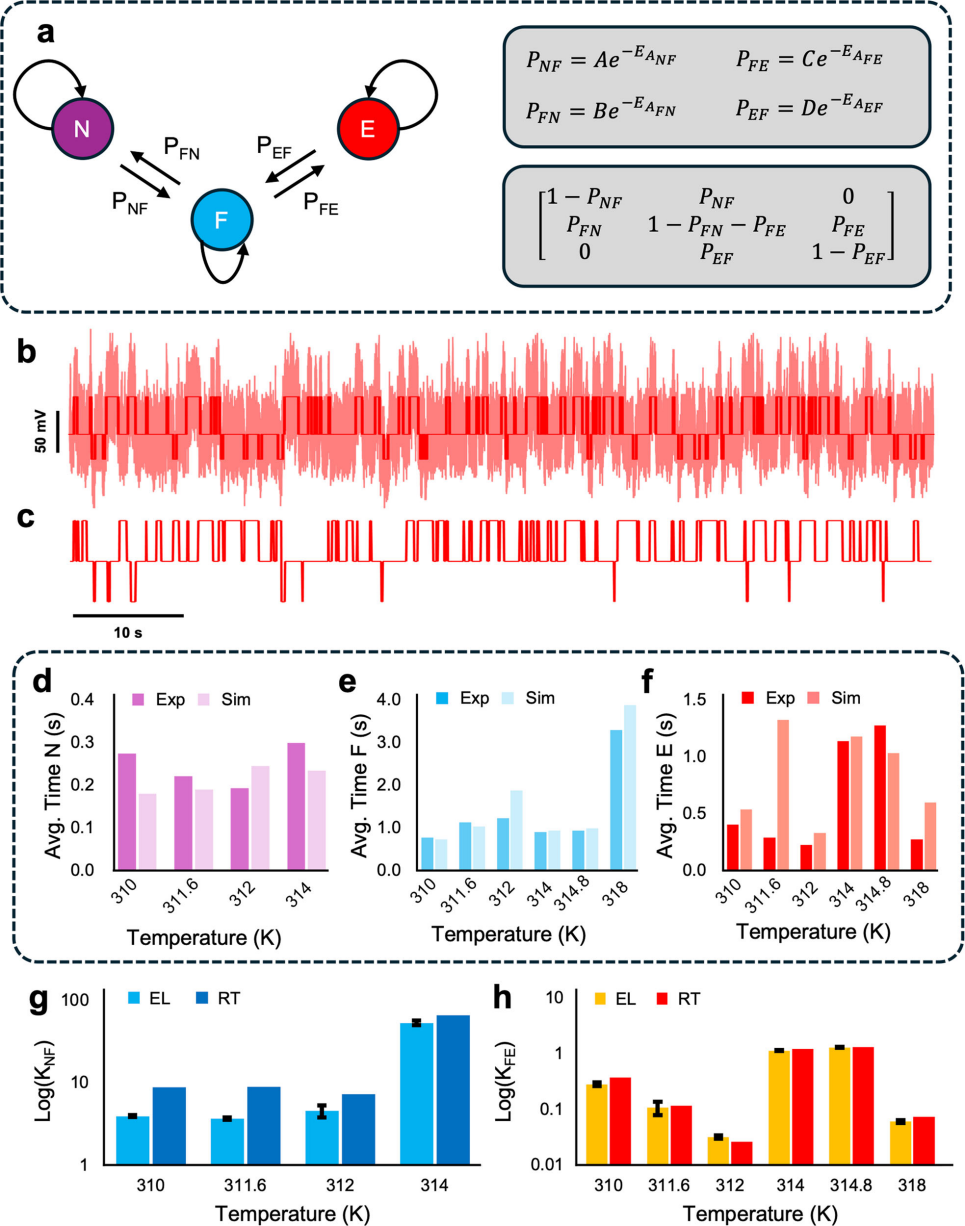
Markov chain simulations of conformational changes. **a** Markov chain representation of conformations and associated probabilities and transition matrix. **b** Time series signal of conformational changes at 310 K, raw data in light red, identified states in dark red. **c** Markov chain simulation of trapping signal using values obtained from energy landscapes. **d** Average residence time of the N state over a range of temperatures measured from experiments and simulations. **e** Average residence time of the F state over a range of temperatures measured from experiments and simulations. **f** Average residence time of the E state over a range of temperatures measured from experiments and simulations. **g** Comparison of thermodynamic equilibrium constant for the N ↔ F pathway. Values obtained via energy landscape (EL) and residence times (RT). **h** Comparison of thermodynamic equilibrium constant for the F ↔ E pathway. Values obtained via energy landscape (EL) and residence times (RT).

We define two pathways in this work, the N ↔ F and F ↔ E. To verify the efficacy of the deconvolution in retrieving accurate biophysical parameters, two other analyses were performed for comparison: Markov chain simulations and residence time ratio analysis. In the Markov chain simulation, the probabilities of each pathway and the transition matrix were defined as in Fig. 4a. Further to this, Kramers’ theory in the viscous damping limit describes the forward flux of the reaction as follows^72^:

4 $${J}_{{\rm{N}}\to {\rm{F}}}=\frac{{\omega }_{{\rm{N}}}}{2\pi }\frac{{\omega }_{{\rm{NF}}}}{\zeta }{e}^{\frac{-{E}_{A{\rm{NF}}}}{{k}_{B}T}},$$

where *ω*_N_ is the curvature of the N energy minimum, *ω*_NF_, is the curvature of the N-F barrier, *ζ* is the friction coefficient, and *E*_*A*NF_ the activation energy going from N to F. The curvatures of the N, F, and E minima as well as the barrier curvatures were obtained directly from the energy landscapes (see Supplementary Information Fig. SI-4). Four probabilities are then defined as:

5 $$\begin{array}{ll}{P}_{NF}=A\cdot \exp (-{E}_{ANF})&{P}_{EF}=C\cdot \exp (-{E}_{AEF})\\ {P}_{FN}=B\cdot \exp (-{E}_{AFN})&{P}_{FE}=D\cdot \exp (-{E}_{AFE}),\end{array}$$

and the individual proportionality constants are obtained via the *P*_*NF*_ proportionality constant and the ratio of curvatures for the barriers and wells:

6 $$B=A\cdot \frac{{\omega }_{{\rm{F}}}}{{\omega }_{{\rm{N}}}},\quad C=A\cdot \frac{{\omega }_{{\rm{FE}}}}{{\omega }_{{\rm{NF}}}}\cdot \frac{{\omega }_{{\rm{F}}}}{{\omega }_{{\rm{E}}}},\quad D=A\cdot \frac{{\omega }_{{\rm{FE}}}}{{\omega }_{{\rm{NF}}}}\cdot \frac{{\omega }_{{\rm{E}}}}{{\omega }_{{\rm{F}}}}$$

In the residence time analysis, the thermodynamic equilibrium constant was obtained from the ratio of the total duration in each state. Namely, ${K}_{NF}=\frac{{\tau }_{F}}{{\tau }_{N}}$ and ${K}_{FE}=\frac{{\tau }_{E}}{{\tau }_{F}}$.

The segments of energy landscapes used for curvature measurements are shown in the Supplementary Information (Fig. SI-4). The proportionality constant *A* was optimised using an unconstrained nonlinear optimisation, given the experimental average residence times as the goal. This is the only free parameter of the model. Simulations were run to achieve a 1000 s time series.

The activation energy is obtained from the full time series data (example shown in Fig. 4b). A k-Means state recognition algorithm was used to find the experimental state switching, also shown in part b. Figure 4c shows 80 s of the corresponding Markov simulation using the activation energies and change in Gibbs free energy from the experimental energy landscape. A comparison of the average residence times for the N, F, and E-state is shown in Fig. 4d–f. After verifying the deconvolution result, the thermodynamic equilibrium constant obtained via the energy landscape was compared with kinetic analysis using the full residence time ratios, seen in Fig. 4g, h.

## Discussion

Through a deconvolution procedure of the time-averaged data, we have presented the energy landscape of monomeric BSA for various temperatures (see Supplementary Information Fig. SI-5). We then reconnected this with the kinetics by using a Markov model with Kramers’ theory transition rates. In this current study, the PSF was determined using a stable version of the same protein; however, in the future, a calibration curve can be obtained, allowing for the extraction of a PSF for any intermediate protein a priori. The PSF arises from the translational and rotational motion of the protein in the trap, and this is measured by the RMSD and the corner frequency in each state. We found negligible (within 6%) changes in RMSD values between each state, implying that the PSF is approximately state independent. Thus we can use a fixed PSF for conformational changes in a single protein, but the dimer retains the linear proportionality to molecular weight for the PSF. Good qualitative agreement was seen between the Markov model and the observe kinetic data. Quantitatively, the average time from the Markov model agreed well with average residence times from the k-means state recognition. There is a notable exception of the E state for *T* = 311.6 K, where the energy landscape of this state does not have a pronounced barrier (see Supplementary Information Fig. SI-5) and so perhaps it was not appropriate to apply the Kramers’ theory for that particular case. The main point of the Markov model is to show that we can use Kramers’ theory to explain the underlying biophysics of the conformational changes, i.e., the time domain data. In adopting a viscous damping Kramers model, only a single free parameter exists. While there was one free parameter for fitting the Kramers’ theory for each temperature, these could be further constrained by Eq. (3) from the curvature of the minima and barriers, assuming the viscous damping is only weakly temperature dependent. Indeed, if we divide the fitting parameter by *ω*_N_*ω*_NF_, the value is almost the same (to within 27%) over the different temperatures tested. So, all the time domain dynamics are represented from energy minima and maxima curvatures in the energy landscape. We believe, therefore, that this is the essential information to explain the biophysics of these conformational changes.

The extracted energy landscape was used to provide the equilibrium constants through Eq. (3), and this agreed quantitatively with the ratio of the residence times from the experimental data. This shows accuracy of the deconvolution in producing changes in Gibbs free energy state minima. We showed the deconvolution robustness by attempting slightly different PSF widths, as shown with the error bars. It is also noted that the different states could have different PSFs, and this could explain some of the differences seen between the modelling and the experiments.

While we have extracted the energy landscapes and related Gibbs free energy values, we can consider what these say about the changes in enthalpy and entropy when going between states. The change in Gibbs free energy is related to the change in enthalpy (Δ*H*) and change in entropy (Δ*S*) of the protein from the following equation:

7 $$\Delta G=\Delta H-T\Delta S.$$

For the N to F transition, we observed that change in Gibbs free energy is almost linearly dependent on temperature. This suggests that it is mainly an entropic variation between the different temperatures. Since the entropy is defined by the available degrees of freedom in each state, we expect that *S* will not change with temperature, but the entropic contribution *T*Δ*S* will vary linearly with temperature, as expressed in Eq. (7). Using this relation, we found for unlabelled BSA Δ*G* of 130 ± 55 − 0.43T ± 0.18 kCal/mol, while FITC labelled BSA gave 150 ± 20 − 0.48T ± 0.07 kCal/mol. We have not found any reports of conformational entropy changes for BSA for the N-F transition for 3 states; however, one work reported for only two states and obtained entropy and enthalpy change values in the same order of magnitude as we find here^58^. For the F to E transition, a simple linear temperature dependence was not observed; indeed, there is a minima in the change of Gibbs free energy with temperature (see Supplementary Information Fig. SI-6) that requires further theoretical support. Also, labelled and unlabelled BSA showed similar but not identical behaviour with temperature changes (see Supplementary Information). Repeatability is shown with good agreement of Gibbs free energy values with measurements over multiple days and DNHs.

The laser power changes the optical force, but the force acts to keep the BSA in the potential minima, where the net force is zero. Therefore, to first approximation, the force changes do not play a role in conformational changes; however, higher order effects, like electrostriction, may have some contribution. Due to the ergodic nature of our observations, electrostriction is expected to not be dominant—the protein transitions between states at all temperatures observed and is not forced into a single particular state.

There is often question of whether trapping is a single protein or an aggregate: using video and photodiode analysis, we showed that the initial trap is of a monomeric protein that forms a dimer when a second protein enters the trap (see Supplementary Information Fig. SI-2 and SI-7). The dynamics found once the dimer was formed in the trap when a second monomer entered matched the observed behaviour seen when a dimer was trapped as a whole. There is a transition from monomer to dimer after a second monomer is trapped. We did not observe the F and E states for the dimer. Previous literature has reported that the large stabilisation energy of dimers comes from intersubunit interactions and is substantially greater than monomeric forms^73^.

The dimer transitions show a typical two-state system: the closed state of the dimer is the natural formation where the two heart shapes are compact and an open state that is more extended and hence has larger polarizability. We have only found structural data for the compact dimer state, from which the polarizability values were calculated (see Supplementary Information). Calculated and extrapolated values for the polarizabilities are shown in Table SI-1 and Fig SI-3a, b.

We have previously shown the ability to track proteins prior to them entering the trap^74^. In the present case, we were able to use this capability to confirm that a second BSA molecule was entering the trap after one was already trapped (see Supplementary Information). Once trapped, we could also use the camera to record the changes in the conformation of the protein in the trap (see Supplementary Information Fig. SI-8), which shows that we will be able to perform the entire measurement in the reflection geometry in the future^75^. This would allow easier integration of microfluidics and external heat control as there would be no concern about the condenser lens path.

In this work, we have used the time dynamics of trapped monomeric and dimeric BSA to uncover the energy landscape of the protein undergoing conformational changes. We used a Markov model with viscous Kramers’ theory to model the observed dynamics from the extracted energy landscape, showing good agreement with the actual observations. We quantified changes in Gibbs free energy between the N, F and E states of BSA, and found changes in enthalpy and entropy between the N and F states to be 132 kcal/mol and 0.43 kcal/mol for unlabelled BSA, and slightly larger values were found for FITC labelled BSA, showing that the impact of labelling is not significant within the precision of this measurement. The F to E transition showed more complicated behaviour. We also observed formation of a dimer in the trap, and found that not only did the dimer exhibit a two state transition, but it also appeared to be stabilised against transitions to the F and E states for each of the subunits. In the future, looking at a fully cross linked version of a protein with known conformational changes could be used to verify specific conformations identified during trapping.

This technique is most interesting to uncover the energy landscape and related biophysics of single proteins because it does not require any modifications to the protein being studied—neither labelling nor tethering. Not only does it make the approach easy to use and useful for assessing the impact of labels, but it also opens the door to studying proteins that are challenging to label reliably, such as intrinsically disordered proteins.

## Methods

### Colloidal lithography of double nanoholes

DNHs were fabricated using a modified approach from past works^76^. Standard microscope slides (Fisherbrand 12-550C, 75 × 50 × 1.0 mm^3^) were cut into thirds using a diamond scribe, cleaned by sonicating in ethanol for ten minutes, and dried under nitrogen. A solution was made of 20 μL of 300 nm polystyrene beads (Alpha Nanotech) and 1 mL of ethanol. Using 10 μL of the polystyrene-ethanol solution, a zig-zag pattern was deposited on the microscope slides and left overnight. Once the ethanol evaporated, the square slides were put in an oxygen plasma machine (Harrick PDC) for 170 s. To ensure reproducibility of aperture size and shape, the slides were etched individually in the same location to ensure consistent plasma coverage. The coated slides were then coated with 7 nm of titanium and 70 nm of gold using a sputter deposition system (Mantis QUBE). Polystyrene was removed from the metal by sonicating upside down in ethanol for 7 min. No surface passivation was used, although we previously investigated the impact of passivation with mPEG-thiol on the same BSA-trapping system and it did not show discernibly different results^34^.

### Trapping solution

A 24 × 60-1 microscope cover glass (Globe Scientific Inc.) was cleaned using isopropyl alcohol and dried under nitrogen. An image spacer (Grace Bio-Labs GBL-654008-100EA) was placed in the centre of the coverslip and 9.37 μL of the solution was pipetted into the spacer. The gold DNH sample was placed on top of the spacer so the gold is in contact with the solution. Bovine Serum Albumin (Fisher MWGF70-1KT) was prepared at 1 mg/mL in 10 μM PBS and stored in 20 μL aliquots at −20 °C.

### Microscope

The laser was collimated out of the fibre to a beam size of 2 mm and passed through a linear polarizer and half-waveplate to control the orientation of polarisation. The laser was further expanded 2 times with a beam expander and was incident on a 100 ×, 1.25 NA oil immersion objective (Nikon E-plan), filling the back of the objective. The transmitted light through the sample was collected by a 10× objective (Nikon MRP70100) and focused onto an avalanche photodiode (Thorlabs APD120A). The laser was under-focused to maximise the transmission for the best sensitivity. The beam spot diameter was measured to be 1580 nm by aligning on an DNH for maximum transmission and moving the piezo-stage until the transmission decreased by half and measuring the distance travelled.

### Software, statistical analysis, and data acquisition

All data analysis was performed using custom python and Matlab code. Data was collected at a sampling rate of 100 kHz. Total number of trapping signals analyzed was 24. The root-mean-squared-deviation (RMSD) was calculated by dividing the same 5 s portion of the trapped signal into sections based on a window length of 5000. The RMSD was then divided by the mean of the trapped signal and an average of all the sections was taken.

### Energy landscapes

Typical experiments consisted of one or two trapping events per day. Each day, trapping was done on a separate sample. It typically took 20 min to obtain trapping, and then the trapping was maintained while spending a few minutes at each laser power (temperature) value. Drift from the microscope stage was accounted for by splitting the files into 20 s segments where state recognition was performed. The segments were then spliced back together with the segment shifted such that the mean of the F-state is consistent throughout the time series. Preprocessing of the data further included dithering with a least significant bit of 0.002441 V to reduce the binning error. MATLAB “histcounts” was used to obtain the probability density function with 100 bins. This was padded evenly with zeros to obtain a length of 128 bins. Using MATLABs “deconvlucy” function, the counts and Gaussian point spread function were deconvolved using 51 iterations and $\sqrt{0.0001}$ damping threshold. The energy landscape was calculated using *U*(*x*) = −1 × *ln*(*counts*). Thermodynamic values were calculated by obtaining the minimum free energy value for each state denoted *M*_*N*_, *M*_*F*_, and *M*_*E*_, and the free energy at the maxima of the saddles denoted *S*_1_ and *S*_2_. The activation energies were calculated by *E*_*N*_ = *S*_1_ − *M*_*N*_, *E*_*NF*_ = *S*_1_ − *M*_*F*_, *E*_*FE*_ = *S*_2_ − *M*_*F*_, and *E*_*F*_ = *S*_2_ − *M*_*E*_. The thermodynamic equilibrium constant was calculated using ${K}_{eq}^{NF}={e}^{-({M}_{F}-{M}_{N})}$ and ${K}_{eq}^{FE}={e}^{-({M}_{E}-{M}_{F})}$.

#### State recognition

State recognition was achieved using a k-means method. The trapping signal was split into 20 s segments. To decrease the effects of the signal noise, the total data sequence was separated into 100 ms slices, with the average of the slice representing the state of the signal. This process worked well when there are only 2 states in the data sequence. The third state often showed up shortly and rarely, so a 2-state 1D k-means method was used to classify the 3-state signal temporarily. Two two-state classifications were performed and then combined to obtain the full three state classification (as detailed in the Supplementary Information).

## Supplementary information

Supplementary Video 1

Supplementary Information

## Data Availability

Data and code is available at https://github.com/nanoplasmonics/EnergyLandscape.

## References

[1] Moerner, W. A dozen years of single-molecule spectroscopy in physics, chemistry, and biophysics. *J. Phys. Chem. B* **106**, 910–927 (2002).

[2] Chung, H. S., McHale, K., Louis, J. M. & Eaton, W. A. Single-molecule fluorescence experiments determine protein folding transition path times. *Science* **335**, 981–984 (2012). 22363011 10.1126/science.1215768 PMC3878298

[3] Chung, H. S. & Eaton, W. A. Protein folding transition path times from single molecule fret. *Curr. Opin. Struct. Biol.* **48**, 30–39 (2018). 29080467 10.1016/j.sbi.2017.10.007 PMC5826754

[4] Gambin, Y. & Deniz, A. A. Multicolor single-molecule fret to explore protein folding and binding. *Mol. Biosyst.* **6**, 1540–1547 (2010). 20601974 10.1039/c003024d PMC3005188

[5] Petrosyan, R., Narayan, A. & Woodside, M. T. Single-molecule force spectroscopy of protein folding. *J. Mol. Biol.* **433**, 167207 (2021). 34418422 10.1016/j.jmb.2021.167207

[6] Schlierf, M., Berkemeier, F. & Rief, M. Direct observation of active protein folding using lock-in force spectroscopy. *Biophys. J.* **93**, 3989–3998 (2007). 17704164 10.1529/biophysj.107.114397 PMC2084248

[7] Sharma, D. et al. Single-molecule force spectroscopy reveals a mechanically stable protein fold and the rational tuning of its mechanical stability. *Proc. Natl Acad. Sci.* **104**, 9278–9283 (2007). 17517616 10.1073/pnas.0700351104 PMC1890485

[8] Gebhardt, J. C. M., Bornschlögl, T. & Rief, M. Full distance-resolved folding energy landscape of one single protein molecule. *Proc. Natl Acad. Sci.* **107**, 2013–2018 (2010). 20133846 10.1073/pnas.0909854107 PMC2836620

[9] Woodside, M. T. & Block, S. M. Reconstructing folding energy landscapes by single-molecule force spectroscopy. *Annu. Rev. Biophys.* **43**, 19–39 (2014). 24895850 10.1146/annurev-biophys-051013-022754 PMC4609573

[10] Woodside, M. T. et al. Direct measurement of the full, sequence-dependent folding landscape of a nucleic acid. *Science* **314**, 1001–1004 (2006). 17095702 10.1126/science.1133601 PMC2656380

[11] Yu, H. et al. Energy landscape analysis of native folding of the prion protein yields the diffusion constant, transition path time, and rates. *Proc. Natl Acad. Sci.* **109**, 14452–14457 (2012). 22908253 10.1073/pnas.1206190109 PMC3437844

[12] Sun, Y. et al. Effect of fluorescently labeling protein probes on kinetics of protein-ligand reactions. *Langmuir* **24**, 13399–13405 (2008). 18991423 10.1021/la802097z PMC2721158

[13] Dietz, M. S., Wehrheim, S. S., Harwardt, M.-L. I., Niemann, H. H. & Heilemann, M. Competitive binding study revealing the influence of fluorophore labels on biomolecular interactions. *Nano Lett.* **19**, 8245–8249 (2019). 31621335 10.1021/acs.nanolett.9b03736

[14] Yin, L. et al. How does fluorescent labeling affect the binding kinetics of proteins with intact cells? *Biosens. Bioelectron.* **66**, 412–416 (2015). 25486538 10.1016/j.bios.2014.11.036 PMC4836836

[15] Liang, F., Guo, Y., Hou, S. & Quan, Q. Photonic-plasmonic hybrid single-molecule nanosensor measures the effect of fluorescent labels on dna-protein dynamics. *Sci. Adv.* **3**, e1602991 (2017). 28560341 10.1126/sciadv.1602991 PMC5446212

[16] Weisgerber, A. W. & Knowles, M. K. Membrane dynamics are slowed for alexa594-labeled membrane proteins due to substrate interactions. *BBA Adv.* **1**, 100026 (2021). 37082018 10.1016/j.bbadva.2021.100026 PMC10074974

[17] Hughes, L. D., Rawle, R. J. & Boxer, S. G. Choose your label wisely: water-soluble fluorophores often interact with lipid bilayers. *PloS ONE* **9**, e87649 (2014). 24503716 10.1371/journal.pone.0087649 PMC3913624

[18] Zanetti-Domingues, L. C., Tynan, C. J., Rolfe, D. J., Clarke, D. T. & Martin-Fernandez, M. Hydrophobic fluorescent probes introduce artifacts into single molecule tracking experiments due to non-specific binding. *PloS ONE* **8**, e74200 (2013). 24066121 10.1371/journal.pone.0074200 PMC3774629

[19] Riback, J. A. et al. Commonly used fret fluorophores promote collapse of an otherwise disordered protein. *Proc. Natl Acad. Sci.* **116**, 8889–8894 (2019). 30992378 10.1073/pnas.1813038116 PMC6500129

[20] Yousefi, A. et al. Structural flexibility and disassembly kinetics of single ferritin molecules using optical nanotweezers. *ACS Nano* **18**, 15617–15626 (2024). 38850556 10.1021/acsnano.4c01221 PMC11191739

[21] Yousefi, A. et al. Optical monitoring of in situ iron loading into single, native ferritin proteins. *Nano Lett.* **23**, 3251–3258 (2023). 37053043 10.1021/acs.nanolett.3c00042 PMC10141409

[22] Ma, G. et al. Optical imaging of single-protein size, charge, mobility, and binding. *Nat. Commun.* **11**, 4768 (2020). 32958747 10.1038/s41467-020-18547-w PMC7505846

[23] Banerjee, A. et al. Influence of point mutations on pr65 conformational adaptability: insights from molecular simulations and nanoaperture optical tweezers. *Sci. Adv.* **10**, eadn2208 (2024). 38820156 10.1126/sciadv.adn2208 PMC11141623

[24] Ying, C. et al. Watching single unmodified enzymes at work arXiv preprint arXiv:2107.06407 (2021). 10.48550/arXiv.2107.06407.

[25] Špačková, B. et al. Label-free nanofluidic scattering microscopy of size and mass of single diffusing molecules and nanoparticles. *Nat. Methods* **19**, 751–758 (2022). 35637303 10.1038/s41592-022-01491-6 PMC9184284

[26] Zhang, P. et al. Label-free imaging of nanoscale displacements and free-energy profiles of focal adhesions with plasmonic scattering microscopy. *ACS Sens.* **6**, 4244–4254 (2021). 34711049 10.1021/acssensors.1c01938 PMC8638434

[27] Thiele, J. C., Pfitzner, E. & Kukura, P. Single-protein optical holography. *Nat. Photon.* **18**, 388–395 (2024).

[28] Baaske, M. D., Asgari, N., Punj, D. & Orrit, M. Nanosecond time scale transient optoplasmonic detection of single proteins. *Sci. Adv.* **8**, eabl5576 (2022). 35030027 10.1126/sciadv.abl5576 PMC8759745

[29] Wu, D. & Piszczek, G. Measuring the affinity of protein-protein interactions on a single-molecule level by mass photometry. *Anal. Biochem.* **592**, 113575 (2020). 31923382 10.1016/j.ab.2020.113575 PMC7069342

[30] Verschueren, D., Shi, X. & Dekker, C. Nano-optical tweezing of single proteins in plasmonic nanopores. *Small Methods* **3**, 1800465 (2019).

[31] Peri, S. S. S. et al. Detection of specific antibody-ligand interactions with a self-induced back-action actuated nanopore electrophoresis sensor. *Nanotechnology* **31**, 085502 (2019). 31675752 10.1088/1361-6528/ab53a7

[32] Juan, M. L., Gordon, R., Pang, Y., Eftekhari, F. & Quidant, R. Self-induced back-action optical trapping of dielectric nanoparticles. *Nat. Phys.* **5**, 915–919 (2009).

[33] Pang, Y. & Gordon, R. Optical trapping of 12 nm dielectric spheres using double-nanoholes in a gold film. *Nano Lett.* **11**, 3763–3767 (2011). 21838243 10.1021/nl201807z

[34] Pang, Y. & Gordon, R. Optical trapping of a single protein. *Nano Lett.* **12**, 402–406 (2012). 22171921 10.1021/nl203719v

[35] Berthelot, J. et al. Three-dimensional manipulation with scanning near-field optical nanotweezers. *Nat. Nanotechnol.* **9**, 295–299 (2014). 24584272 10.1038/nnano.2014.24

[36] Kerman, S. et al. Raman fingerprinting of single dielectric nanoparticles in plasmonic nanopores. *Nanoscale* **7**, 18612–18618 (2015). 26490057 10.1039/c5nr05341b

[37] Jensen, R. A. et al. Optical trapping and two-photon excitation of colloidal quantum dots using bowtie apertures. *ACS Photon.* **3**, 423–427 (2016).

[38] Raza, M. U., Peri, S. S. S., Ma, L.-C., Iqbal, S. M. & Alexandrakis, G. Self-induced back action actuated nanopore electrophoresis (sane). *Nanotechnology* **29**, 435501 (2018). 30073973 10.1088/1361-6528/aad7d1

[39] Yoo, D. et al. Low-power optical trapping of nanoparticles and proteins with resonant coaxial nanoaperture using 10 nm gap. *Nano Lett.* **18**, 3637–3642 (2018). 29763566 10.1021/acs.nanolett.8b00732

[40] Kotnala, A., Kollipara, P. S., Li, J. & Zheng, Y. Overcoming diffusion-limited trapping in nanoaperture tweezers using opto-thermal-induced flow. *Nano Lett.* **20**, 768–779 (2019). 31834809 10.1021/acs.nanolett.9b04876 PMC6952578

[41] Yoon, S. J. et al. Hopping of single nanoparticles trapped in a plasmonic double-well potential. *Nanophoton.* **9**, 4729–4735 (2020).

[42] Jiang, Q., Rogez, B., Claude, J.-B., Baffou, G. & Wenger, J. Quantifying the role of the surfactant and the thermophoretic force in plasmonic nano-optical trapping. *Nano Lett.* **20**, 8811–8817 (2020). 33237789 10.1021/acs.nanolett.0c03638

[43] Kotsifaki, D. G., Truong, V. G. & Nic Chormaic, S. Fano-resonant, asymmetric, metamaterial-assisted tweezers for single nanoparticle trapping. *Nano Lett.* **20**, 3388–3395 (2020). 32275440 10.1021/acs.nanolett.0c00300

[44] Li, N. et al. Algorithm-designed plasmonic nanotweezers: quantitative comparison by theory, cathodoluminescence, and nanoparticle trapping. *Adv. Optical Mater.* **9**, 2100758 (2021).

[45] Yang, W., van Dijk, M., Primavera, C. & Dekker, C. Fib-milled plasmonic nanoapertures allow for long trapping times of individual proteins. *iScience* **24**, 103237 (2021). 34746702 10.1016/j.isci.2021.103237 PMC8551080

[46] Wu, B. et al. Directivity-enhanced detection of a single nanoparticle using a plasmonic slot antenna. *Nano Lett.* **22**, 2374–2380 (2022). 35285643 10.1021/acs.nanolett.1c04949

[47] Hong, C. & Ndukaife, J. C. Scalable trapping of single nanosized extracellular vesicles using plasmonics. *Nat. Commun.* **14**, 4801 (2023). 37558710 10.1038/s41467-023-40549-7 PMC10412615

[48] Rosenoer, V. M., Oratz, M. & Rothschild, M. A. *Albumin: Structure, function and uses* (Elsevier, 2014).

[49] Baler, K. et al. Electrostatic unfolding and interactions of albumin driven by ph changes: a molecular dynamics study. *J. Phys. Chem. B* **118**, 921–930 (2014). 24393011 10.1021/jp409936v PMC3983335

[50] Peters, T. Jr., Serum albumin. *Adv. Protein Chem.* **37**, 161–245 (1985). 3904348 10.1016/s0065-3233(08)60065-0

[51] Fanali, G. et al. Human serum albumin: from bench to bedside. *Mol. Asp. Med.* **33**, 209–290 (2012). 10.1016/j.mam.2011.12.002 22230555

[52] Carter, D. C. et al. Three-dimensional structure of human serum albumin. *Science* **244**, 1195–1198 (1989). 2727704 10.1126/science.2727704

[53] Dockal, M., Carter, D. C. & Ruker, F. Conformational transitions of the three recombinant domains of human serum albumin depending on ph. *J. Biol. Chem.* **275**, 3042–3050 (2000). 10652284 10.1074/jbc.275.5.3042

[54] Sadler, P. J. & Tucker, A. ph-induced structural transitions of bovine serum albumin: Histidine pka values and unfolding of the n-terminus during the n to f transition. *Eur. J. Biochem.* **212**, 811–817 (1993). 8462552 10.1111/j.1432-1033.1993.tb17722.x

[55] Michnik, A., Michalik, K. & Drzazga, Z. Stability of bovine serum albumin at different ph. *J. Therm. Anal. Calorim.* **80**, 399–406 (2005).

[56] Bloomfield, V. The structure of bovine serum albumin at low pH. *Biochemistry* **5**, 684–689 (1966). 5940950 10.1021/bi00866a039

[57] Chi, Z. et al. Investigation on the conformational changes of bovine serum albumin in a wide pH range from 2 to 12. *Spectrosc. Lett.* **51**, 279–286 (2018).

[58] Bian, L., Wu, D. & Hu, W. Temperature-induced conformational transition of bovine serum albumin in neutral aqueous solution by reversed-phase liquid chromatography. *Biomed. Chromatogr.* **28**, 295–301 (2014). 24037907 10.1002/bmc.3020

[59] Farruggia, B. & Picó, G. A. Thermodynamic features of the chemical and thermal denaturations of human serum albumin. *Int. J. Biol. Macromol.* **26**, 317–323 (1999). 10628532 10.1016/s0141-8130(99)00054-9

[60] Picó, G. A. Thermodynamic features of the thermal unfolding of human serum albumin. *Int. J. Biol. Macromol.* **20**, 63–73 (1997). 9110186 10.1016/s0141-8130(96)01153-1

[61] Borzova, V. A. et al. Kinetics of thermal denaturation and aggregation of bovine serum albumin. *PloS ONE* **11**, e0153495 (2016). 27101281 10.1371/journal.pone.0153495 PMC4839713

[62] Mitra, R. K., Sinha, S. S. & Pal, S. K. Hydration in protein folding: thermal unfolding/refolding of human serum albumin. *Langmuir* **23**, 10224–10229 (2007). 17711315 10.1021/la7014447

[63] Bhattacharya, A., Prajapati, R., Chatterjee, S. & Mukherjee, T. K. Concentration-dependent reversible self-oligomerization of serum albumins through intermolecular *β*-sheet formation. *Langmuir* **30**, 14894–14904 (2014). 25409497 10.1021/la5034959

[64] Chowdhury, R., Chattoraj, S., Mojumdar, S. S. & Bhattacharyya, K. Fret between a donor and an acceptor covalently bound to human serum albumin in native and non-native states. *Phys. Chem. Chem. Phys.* **15**, 16286–16293 (2013). 23999556 10.1039/c3cp52296b

[65] Shaw, A. K. & Pal, S. K. Spectroscopic studies on the effect of temperature on ph-induced folded states of human serum albumin. *J. Photochem. Photobiol. B: Biol.* **90**, 69–77 (2008). 10.1016/j.jphotobiol.2007.11.003 18178096

[66] Xu, Z., Song, W. & Crozier, K. B. Direct particle tracking observation and brownian dynamics simulations of a single nanoparticle optically trapped by a plasmonic nanoaperture. *ACS Photon.* **5**, 2850–2859 (2018).

[67] Jiang, Q., Rogez, B., Claude, J.-B., Baffou, G. & Wenger, J. Temperature measurement in plasmonic nanoapertures used for optical trapping. *ACS Photon.* **6**, 1763–1773 (2019).

[68] Toodeshki, E. H., Frencken, A. L., van Veggel, F. C. & Gordon, R. Thermometric analysis of nanoaperture-trapped erbium-containing nanocrystals. *ACS Photon.* **11**, 1390–1395 (2024). 10.1021/acsphotonics.3c00467 PMC11027910 38645996

[69] Booth, L. S. et al. Modelling of the dynamic polarizability of macromolecules for single-molecule optical biosensing. *Sci. Rep.* **12**, 1995 (2022). 35132077 10.1038/s41598-022-05586-0 PMC8821610

[70] Wang, J. et al. icn3d, a web-based 3d viewer for sharing 1d/2d/3d representations of biomolecular structures. *Bioinformatics* **36**, 131–135 (2020). 31218344 10.1093/bioinformatics/btz502 PMC6956771

[71] Wheaton, S. & Gordon, R. Molecular weight characterization of single globular proteins using optical nanotweezers. *Analyst* **140**, 4799–4803 (2015). 25739349 10.1039/c5an00026b

[72] Hänggi, P., Talkner, P. & Borkovec, M. Reaction-rate theory: fifty years after kramers. *Rev. Mod. Phys.* **62**, 251 (1990).

[73] Neet, K. E. & Timm, D. E. Conformational stability of dimeric proteins: quantitative studies by equilibrium denaturation. *Protein Sci.* **3**, 2167–2174 (1994). 7756976 10.1002/pro.5560031202 PMC2142765

[74] Peters, M., McIntosh, D., Branzan Albu, A., Ying, C. & Gordon, R. Label-free tracking of proteins through plasmon-enhanced interference. *ACS Nanosci. Au* **4**, 69–75 (2023). 38406310 10.1021/acsnanoscienceau.3c00045 PMC10885339

[75] Khosravi, B. & Gordon, R. Reflection mode optical trapping using polarization symmetry breaking from tilted double nanoholes. *Opt. Express* **31**, 2621–2627 (2023). 36785271 10.1364/OE.480802

[76] Ravindranath, A. L., Shariatdoust, M. S., Mathew, S. & Gordon, R. Colloidal lithography double-nanohole optical trapping of nanoparticles and proteins. *Opt. Express* **27**, 16184–16194 (2019). 31163802 10.1364/OE.27.016184

[77] Carter, D. C. & Ho, J. X. Structure of serum albumin. *Adv. Protein Chem.* **45**, 153–203 (1994). 8154369 10.1016/s0065-3233(08)60640-3

