# Supplementary material for: Energy landscape of conformational changes for a single unmodified protein
Source: NPJ Biosens. 2024 Nov 6;1(1):14. doi: 10.1038/s44328-024-00014-x (PMC11541220; doi:10.1038/s44328-024-00014-x)
Supplement: Supplementary file 2 — Supplementary Information [file 44328_2024_14_MOESM2_ESM.pdf]

Supplementary Information: Energy Landscape of Conformational Changes for a Single Unmodified Protein

Matthew Peters[1,2], Tianyu Zhao[1,2], Sherin George[1,2], Viet Giang Truong[3], Síle Nic Chormaic[3], Cuifeng Ying[4], René A. Nome[5], and Reuven Gordon[1,2,*]

[1]Department of Electrical Engineering, University of Victoria, Victoria, V8W 2Y2, British Columbia, Canada
[2]Center for Advanced Material & Related Technologies, University of Victoria, Victoria, V8W 2Y2, British Columbia, Canada
[3]Okinawa Institute of Science and Technology Graduate University, Onna, Okinawa, 904-0495, Japan
[4]Advanced Optics and Photonics Laboratory, Department of Engineering, School of Science & Technology, Nottingham Trent University, Nottingham Trent, NG11 8NS, England
[5]Institute of Chemistry, State University of Campinas, Campinas, Brazil
[*]rgordon@uvic.ca

ABSTRACT

COMSOL simulations, dimer dynamics, polarizability of a single protein, trapping events and energy landscapes, F↔E pathways thermodynamics, plasmon enhanced imaging, state recognition.

1 COMSOL simulations

COMSOL simulations of the local heating in a DNH illuminated by a 980 nm laser with 1 mW intensity show the gold thickness dependence on heating in Fig. 1a. Fig. 1b shows the temperature increase for increasing laser power for a 70 nm gold film illuminated by a 980 nm laser.

We used a commercial finite element modeling COMSOL 6.0 package for the simulations. A 3D model was established to solve the electromagnetic (EM) and heat transfer (HT) problems. The modeling process was governed by differential equations describing the full-wave EM and HT physics, and their coupling phenomena. The 3D EM domain was set at 2.0×2.0×2.0 μm cube, consisting of a single DNH at the center of the unit cell, and illuminating the water-side by a laser with a Gaussian intensity distribution. The focal-spot radius was set at $w_0 = 0.61\lambda/NA$, where λ was the wavelength of the incident laser light and NA = 1.25 was the numerical aperture of the focusing objective lens. Scattering boundary conditions were used on all outer EM domain's boundaries. This scattering boundary allowed EM radiation to propagate out of the EM domain without reflection. Subsequently, this full-EM domain was placed inside a larger 100×100×40 μm (Width, Depth, Height) HT domain. A prescribed ambient temperature of 293.15 K was set at the HT boundaries for solving the heat transfer physics.

The input Gaussian beam was polarized parallel to the y-direction. The distribution of the electric field (E-field) was calculated by solving numerically the scattering problem using the time-independent Maxwell's vector wave equation. Once the E-field distribution in the full-EM domain was found, the heat source density was then obtained by $q_i(\mathbf{r}) = 1/2Re[\mathbf{J}\cdot\mathbf{E}*]$, where J is induced-current density, Re represents the real part, and * is the complex conjugate. Finally, the total heat power, $\mathbf{Q_{tot}} = \oint q_i(\mathbf{r})d^3\mathbf{r}$, was observed by taking the sum of the heat power generated by each mesh element, and serves as a total power dissipation heat over the 3D volume of the coupled EM and HT modeling system. The model required 0.55 TB of memory to perform the calculation. Calculation of each thermal heating data point took approximately 5 hours of machine time on the OIST Graduate University computing cluster.

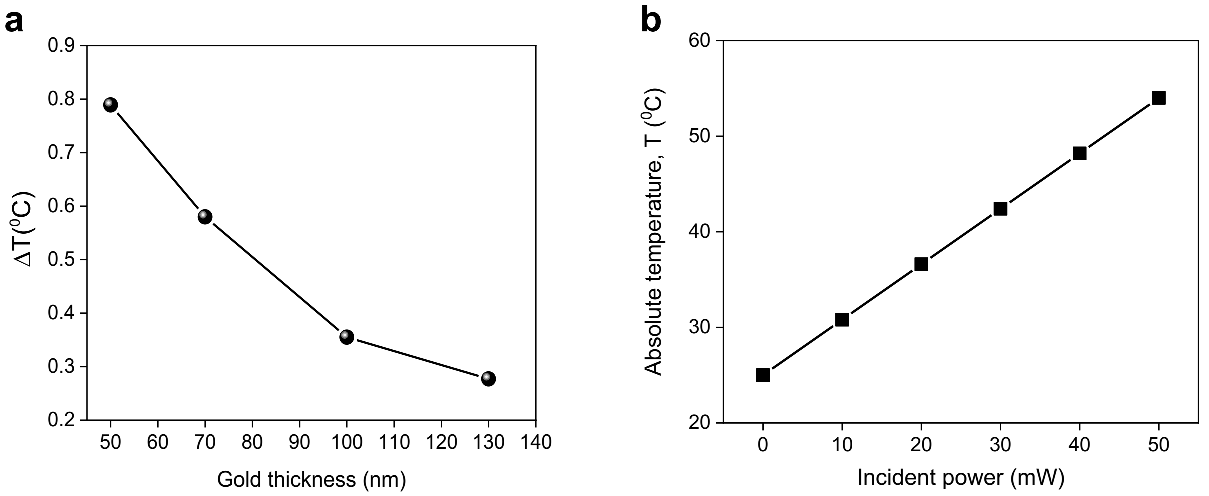

Supplementary Figure 1. (a) Change in local temperature for different gold film thicknesses for an incident 980 nm laser with 1 mW power. (b) Absolute local temperature for increasing incident laser power for 70 nm thick gold film.

2 Dimer Dynamics

Dimer trapping occurred in 2 out of 13 trapping events. A special case is presented here, however, where monomeric BSA was first trapped and a second monomeric BSA entered the trap, forming a dimer, as shown in Fig. 2a-b. The structure of the monomer and dimer are shown in Fig. 2c. The dimeric BSA was observed to have limited conformations: 3 states were observed in monomeric BSA while only 2 states were observed for dimeric BSA. We attribute these two states to a “closed" (C) and “open" (O) form of the dimer due to a hinging mechanism at the binding point[?,?]. While the C-dimer and E-dimer have similar transmission levels, they have significantly different RMSD values (by a factor of 1.9) and time constants so they can easily be distinguished. In the video analysis, we see the second monomer diffusing into the trap; however, we do not consider that it is diffusing out of the trap as it is not evident in video analysis (see SI movie 1). The average molecular polarizability of monomeric BSA in the N-state is 5400 $Å^3$ while of dimeric BSA in the C-state is 9971 $Å^3$. Similar to the monomer, the energy landscape was obtained from the PDF of the trap, seen in Fig. 2d for 316 K.

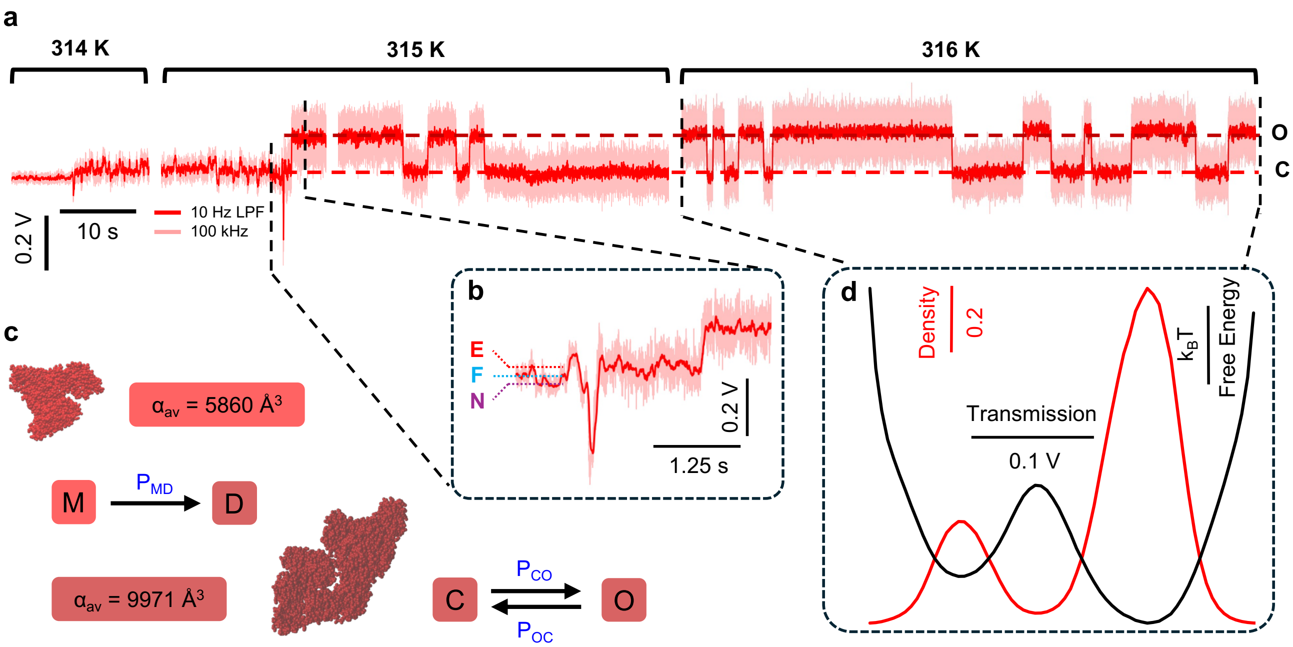

Supplementary Figure 2. (a) Transmission through the DNH at different temperatures showing the initial trap of a monomer and subsequent second trap forming a dimer two-state system. (b) Zoom-in of second trap and dimer formation. (c) Reaction mechanism for the monomer to dimer formation with average molecular polarizabilities calculated. Protein visualizations using PDB: 3V03 for the monomer and PDB: 6QS9 for the dimer. (d) Energy landscape and probability density function for dimeric BSA at 316 K.

3 Polarizability measurement of a single protein

The polarizability of the trapped protein can be obtained by via a calibration curve of known polarizabilities and the experimental root mean square deviation of the trap signal. Fig. 3a shows the polarizabilities of the monomeric form of BSA. Fig. 3b shows the values for the dimeric form. Tab. 1 shows the calculated and extracted polarizability values.

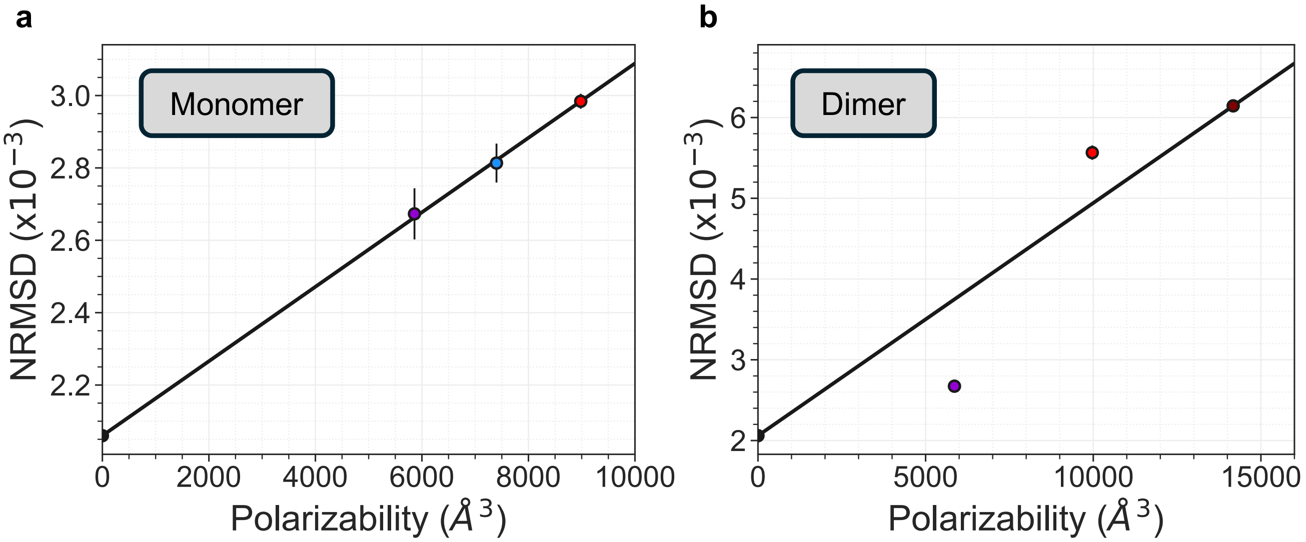

Supplementary Figure 3. Normalized root mean square deviation of the trapping laser signal as a function of polarizability for (a) monomer (black: laser, purple: N, blue: F, and red: E) and (b) dimer (black: laser, purple: N, red: C, maroon: O).

Supplementary Table 1. Polarizability calculation for monomer and dimer conformations from trapping signal RMSD values.

**Conformation**	**RMSD** ($\times 10^{-3}$)	**Calculated Polarizability** (^{3})	**Extracted Polarizability** (^{3})
Laser	2.06 $\pm$ 0.011	0	0
N	2.67 $\pm$ 0.050	5860	5958 $\pm$ 686
F	2.81 $\pm$ 0.049	7400	7322 $\pm$ 523
E	2.98 $\pm$ 0.019		8982 $\pm$ 201
C	5.57 $\pm$ 0.086	9971	
O	6.14 $\pm$ 0.049		14173 $\pm$ 4329

4 Trapping events

Segments of the energy landscape used for obtaining curvatures for the N, F, and E-wells as well as the NF and FE barriers are shown for each temperature in Fig. 4.

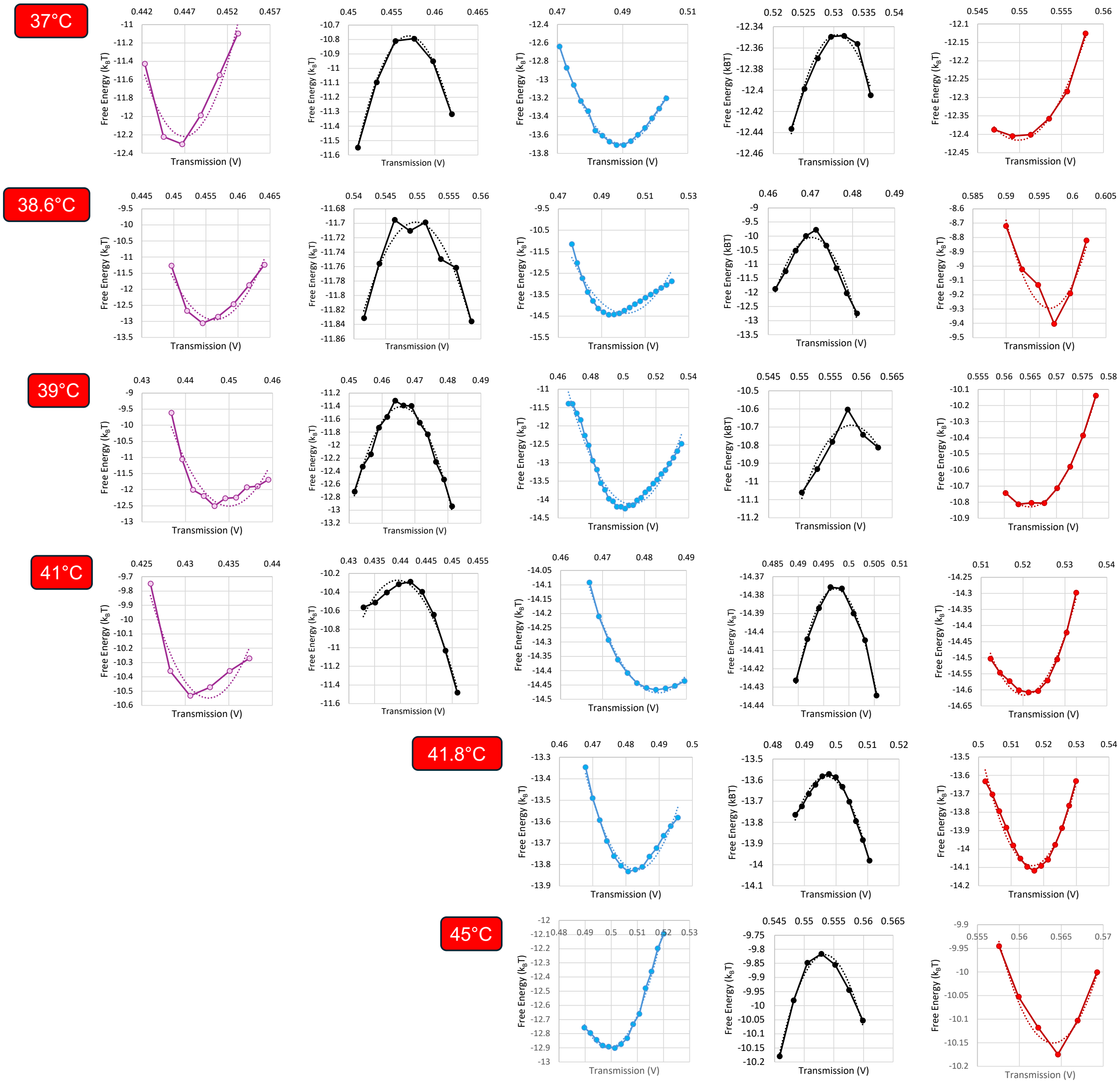

Supplementary Figure 4. Segments of energy landscapes used for curvature measurements. Solid line is the energy landscape, dotted line is a second order polynomial fit.

All trapping events of unlabelled BSA and the corresponding energy landscapes with varying PSF widths are shown in Fig. 5.

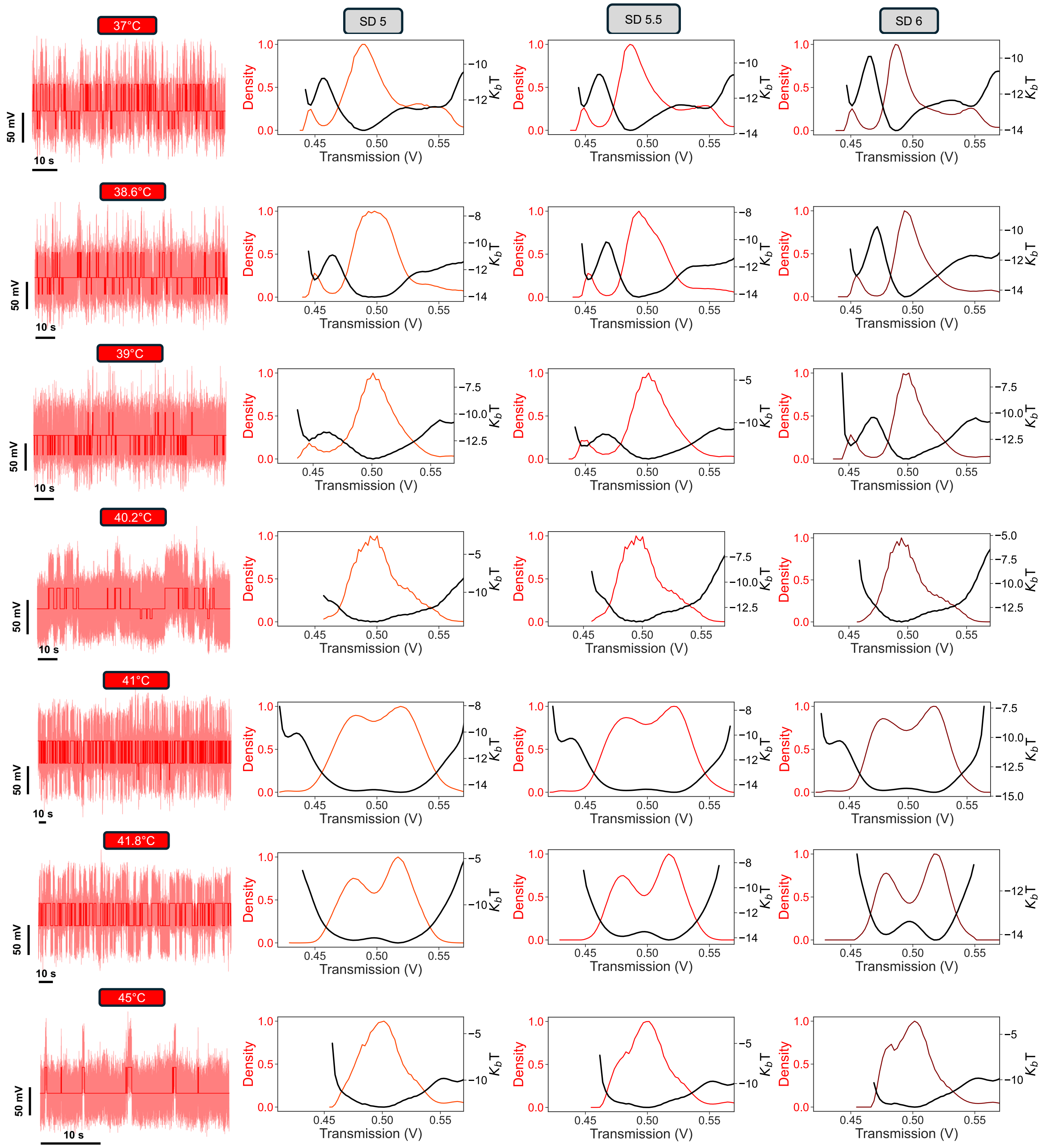

Supplementary Figure 5. Trapping events and energy landscapes with varying point spread function widths

5 Fluorescent label impact

The thermodynamic parameters for labelled and unlabelled BSA for the F↔E pathway is shown in Fig. 6.

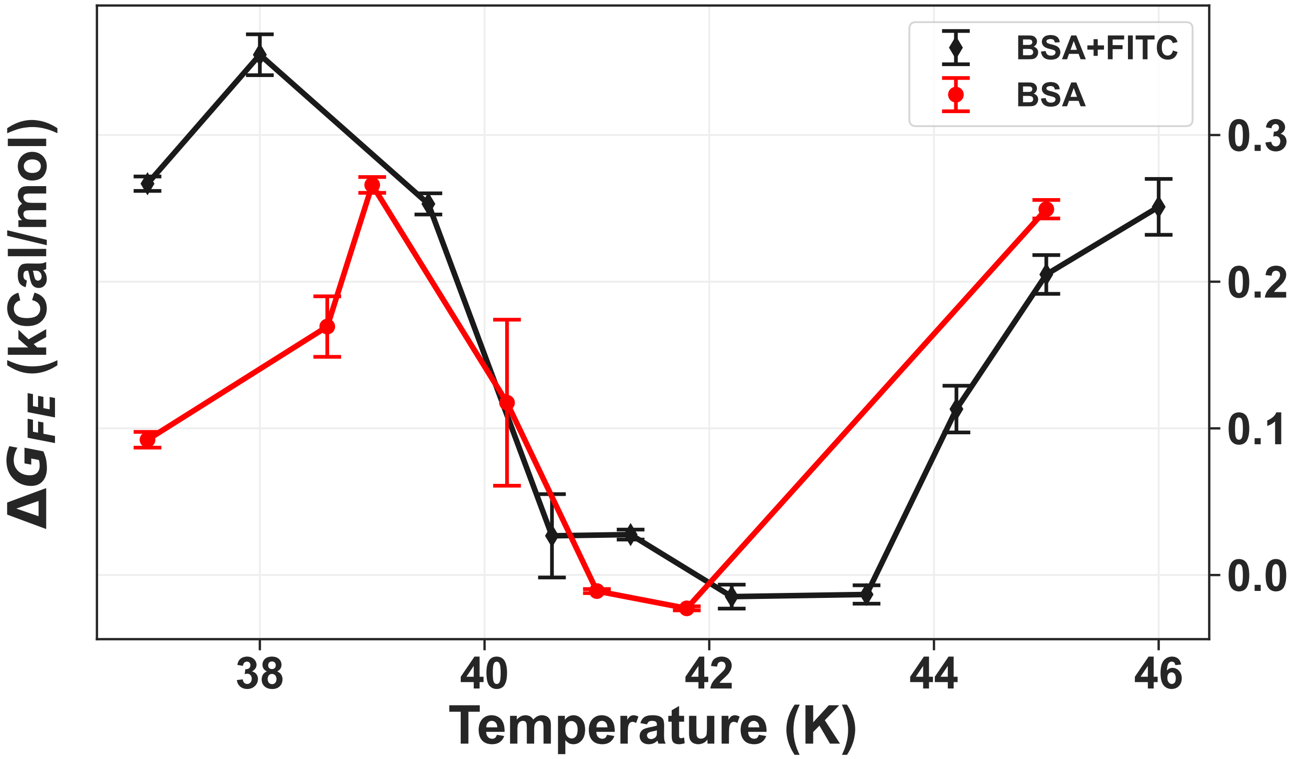

Supplementary Figure 6. Change in Gibbs free energy for labelled and unlabelled BSA for the F↔E pathway.

6 Plasmon enhanced imaging

Previous work has shown that the plasmonic field can provide an enhanced interferometric signal able to image single proteins as they approach the trap and when trapped. Dimer formation is visible in the video information, a monomeric BSA can be seen entering the trap (as verified by corner frequency measurements of APD signal) and a second monomeric BSA is tracked entering the trap. The APD traces of this event are shown in Fig. 3a while the video tracking is shown in Fig. 7.

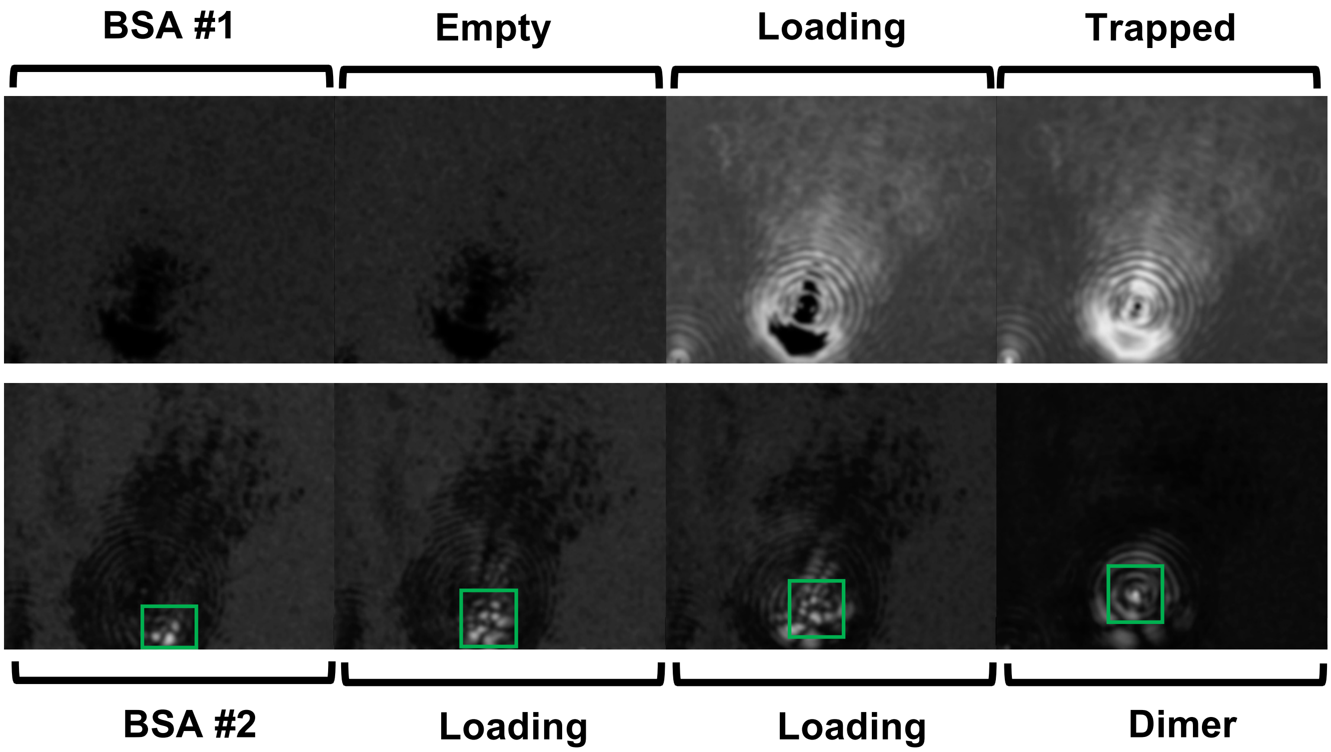

Supplementary Figure 7. Differential imaging of monomeric BSA being trapped, followed by a second BSA being trapped, forming a dimer.

Here we show that once trapped, the intensity of the reflected light can be used to "see" the conformational changes. The intensity of a single pixel from the center of the frame is recorded over time and synced with the APD. Fig. 8 shows that despite a more than 6000× slower sampling rate, the general "picture" of the conformational landscape is preserved in the light intensity. While faster dynamics such as transition times and failed transitions are inaccessible, this should be resolvable with a sufficiently high speed camera.

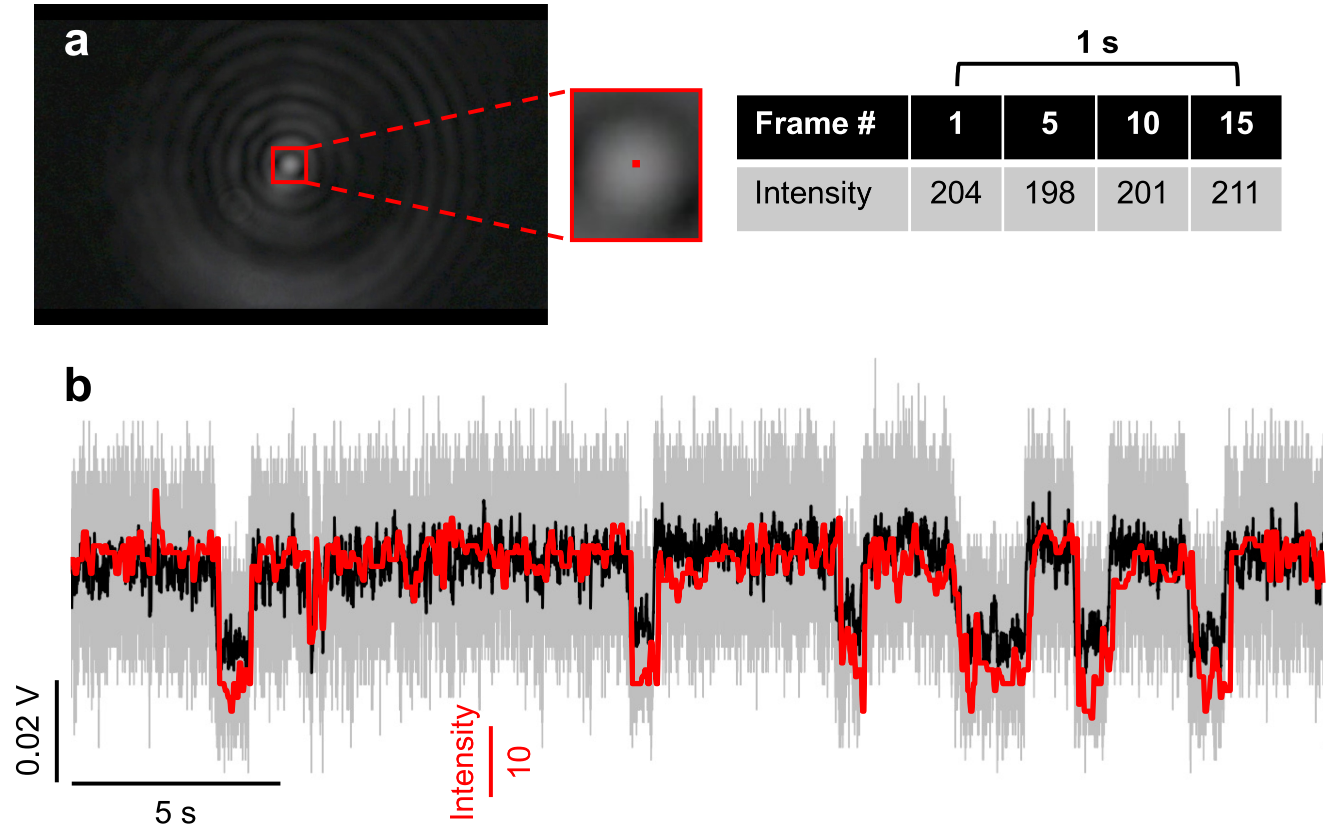

Supplementary Figure 8. (a) Typical frame of a video recorded during trapping. Highlighted region shows the centre lobe of the diffraction pattern and single pixel intensity tracking. (b) Overlay of synced avalanche photodiode measurement at 100 kHz and pixel intensity at 15 Hz.

7 State recognition

A step-by-step process for the state recognition algorithm is shown in Fig. 9.

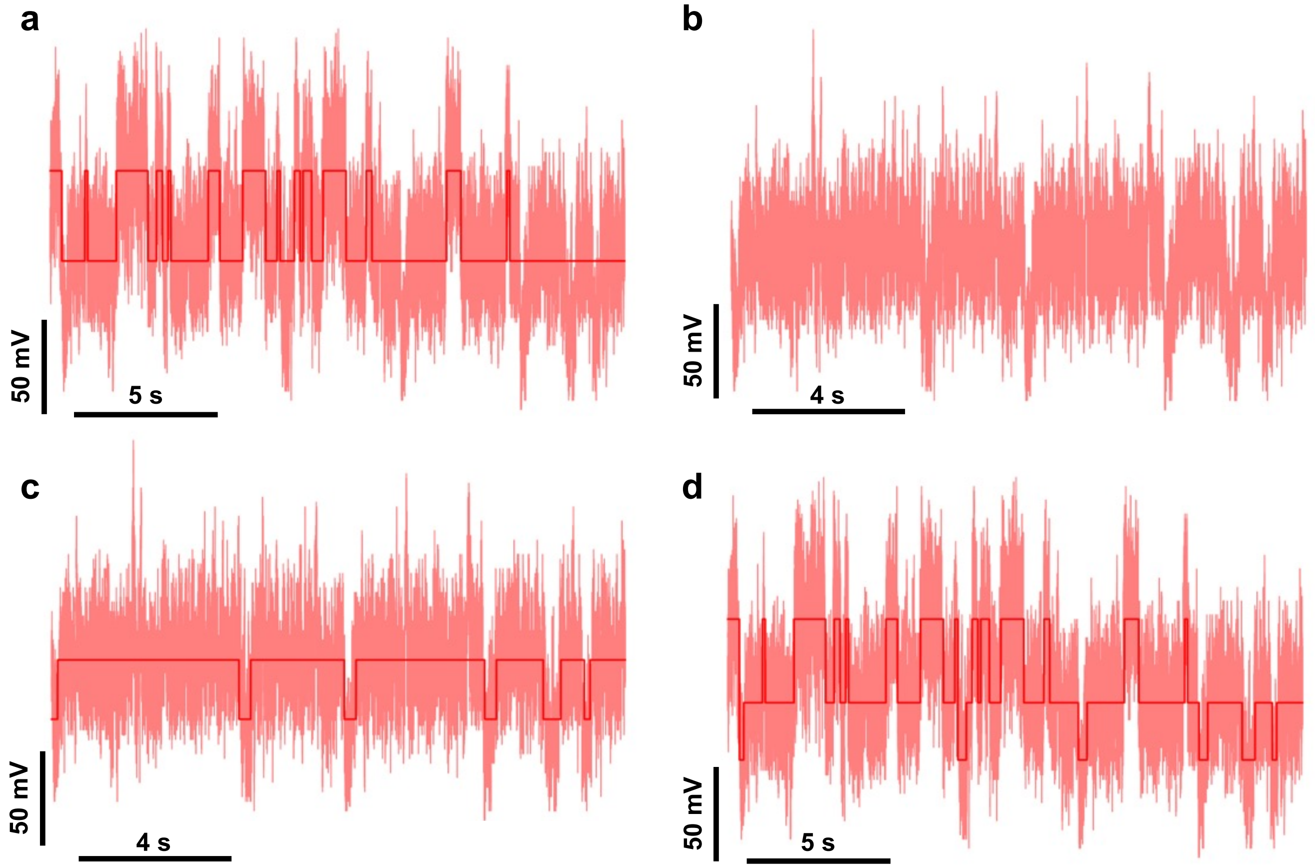

Supplementary Figure 9. (a)Two state recognition on a three state system using k-means state recognition. (b) Removal of upper state. (c) Two state recognition on lower and middle state. (d) Recombination time series and state recognition.
